# Coexistence and distribution patterns of *Anastrepha dentata* and *Anastrepha pallens* (Diptera: Tephritidae) associated with fruits of *Sideroxylon celastrinum* (Sapotaceae) along an altitudinal gradient in East-central Mexico

**DOI:** 10.3389/finsc.2026.1806523

**Published:** 2026-04-20

**Authors:** Maurilio López-Ortega, Jaime C. Piñero, Diego M. Gómez-González, Armando J. Martínez, Vicente Hernández-Ortiz

**Affiliations:** 1 Instituto de Biotecnología y Ecología Aplicada (INBIOTECA), Universidad Veracruzana, Veracruz, Mexico; 2 Stockbridge School of Agriculture, University of Massachusetts, Amherst, MA, United States; 3 Instituto de Neuroetología, Universidad Veracruzana, Veracruz, Mexico; 4 Red de Interacciones Multitróficas, Instituto de Ecología A.C., Veracruz, Mexico

**Keywords:** altitudinal segregation, host-plant association, interactions, natural history, new record, seed-feeding insects

## Abstract

We present the first confirmed host-plant association of *Anastrepha dentata* (Stone) (Diptera: Tephritidae) with *Sideroxylon celastrinum* (Sapotaceae) and provide new insights into its coexistence and altitudinal segregation with *Anastrepha pallens* (Coquillett) in central Veracruz, Mexico. Multi-year surveys across elevations ranging from 270 to 720 m revealed consistent partitioning, with *A. dentata* dominating higher elevations and *A. pallens* prevailing in lowland areas, while both species co-occurred at intermediate elevations. Laboratory rearing demonstrated a strict one-larva-per-fruit pattern and species-specific differences in fruit selection. Canonical discriminant analysis showed that fruit length (not fruit weight), was the primary morphological trait associated with infestation by *A. pallens*, a novel pattern not previously documented for this host-fruit fly system. Developmental timing also differed between species, with *A. dentata* exhibiting slower development at cooler, high-elevation sites and *A. pallens* developing more rapidly in warm lowland environments. Parasitism was minimal, indicating little parasitoid impact on either fly species. Together, these results highlight the combined influence of environmental filtering, fruit morphological traits, and microclimatic variation in structuring the distribution and coexistence of these species. This work expands the natural history of *A. dentata* and *A. pallens* and advances our understanding of ecological differentiation within Neotropical fruit fly communities.

## Highlights

- We document the first confirmed host-plant record of *Anastrepha dentata*.
- *Anastrepha dentata* and *A. pallens* coexist on the same host fruit but segregate along an altitudinal gradient.
- Elevation and microclimatic conditions appear to mediate species dominance and developmental timing.
- This study expands ecological and evolutionary understanding of host use and niche differentiation within *Anastrepha*.

## Introduction

The genus *Anastrepha* Schiner is endemic to the Americas and currently comprises approximately 325 recognized species (1), being the largest genus of fruit flies occurring in tropical and subtropical environments, from south of the USA throughout Mexico, Central and South America, and the Caribbean Islands (2).

To date, host plants have been identified for 148 *Anastrepha* species, which are collectively associated with 494 plant species belonging to 63 botanical families (3). However, the host plant associations of many of these species remain poorly documented. Among these are members of the Sapotaceae family, which are ecologically important components of Neotropical forests and are valued for their economic significance, ethnobotanical uses, latex production, and timber potential (4). Their fruits support the highest number of local *Anastrepha* interactions than any other host plant family (e.g., 3, 5, 6), making them particularly relevant for studies on host specialization, resource use, and behavioural ecology (7).

Host use patterns reveal trophic specialization and niche partitioning, which facilitate the coexistence of closely related species. For example, the Mexican fruitfly, *Anastrepha ludens* (Loew), infests the fruits of *Citrus* spp. (Rutaceae), whereas *Anastrepha obliqua* (Macquart) primarily feeds on native *Spondias* species, however, both may utilize mango (*Mangifera indica* L.) (Anacardiaceae) when it is available. Similarly, *Anastrepha fraterculus* (Wiedemann) and *Anastrepha striata* Schiner are found infesting fruits of *Psidium guajava* L. (Myrtaceae), and sometimes both could be found occupying a single fruit (8). Early research by Baker et al. (9) reported similar tissue-partitioning exploitation in fruits of *Pouteria campechiana* (Kunth) Baehni (Sapotaceae) by *Anastrepha sagittata* (Stone) and *Anastrepha serpentina* (Wiedemann). Further studies on fruits of this host plant showed that *Anastrepha hamata* (Loew) feed on the seeds, while *A. serpentina* fed on the pulp, thus allowing both to share the same host without direct competition (10). The diversification and ecological success of fruit flies in the genus *Anastrepha* are largely driven by evolutionary adaptations that enable the exploitation of internal fruit tissues, particularly pulp and seeds. These morphological, physiological, behavioral, and genomic traits promote host specialization and larval development within plant tissues (1, 11, 12), underscoring the importance of fruit fly–host plant interactions and the need to better understand larval feeding niches and coexistence mechanisms.

In Mexico, there are historical records for 37 species of *Anastrepha* (13), and further additional reports of three other species, *Anastrepha tehuacana* Norrbom (14), *Anastrepha furcata* Lima (15), and an undescribed *Anastrepha* species feeding on *Pouteria reticulata* (Engl.) Eyma (Sapotaceae) (6). This increases to 40 species recorded in the country. All Mexican species belong to 16 taxonomic species groups (*sensu* 1), which are associated with a variety of climatic conditions, topographical features, and biogeographical locations connecting the Nearctic and Neotropical regions (6, 13).

In this context, *Anastrepha pallens* (Coquillett) has been recorded in 14 states of Mexico, along the coastal Pacific tropical forests from Sonora to Chiapas, and also in the states of Tamaulipas and Veracruz on the Gulf of Mexico (13, 16). In addition, it has also been reported in Honduras (17). This fruit fly is known to use fruits of *Sideroxylon celastrinum* (Kunth) T.D. Pennington (Sapotaceae) as unique host plant (18). To date, no specific host plant has been identified for *Anastrepha dentata* (Stone), since it has only been recorded from trapping surveys in Mexico, including Aguascalientes, Jalisco, Morelos, Sinaloa, and Veracruz (10, 16, 19b). It has also reported from southern Texas (USA) (19), making this the first documented report of its host plant.

This study involved a multi-year investigation conducted along an elevational gradient at various locations in the mountains of “Sierra Madre Oriental” in Mexico. We assessed infestation rates in wild fruits of *S. celastrinum*, on which two conspecifics, *A. dentata* and *A. pallens* coexist. Furthermore, we explored host-use patterns across the altitudinal gradient to understand the ecological implications of both species coexisting via exploitation of this wild resource.

## Materials and methods

### Study area and host plant sampling

**Figure 1:**
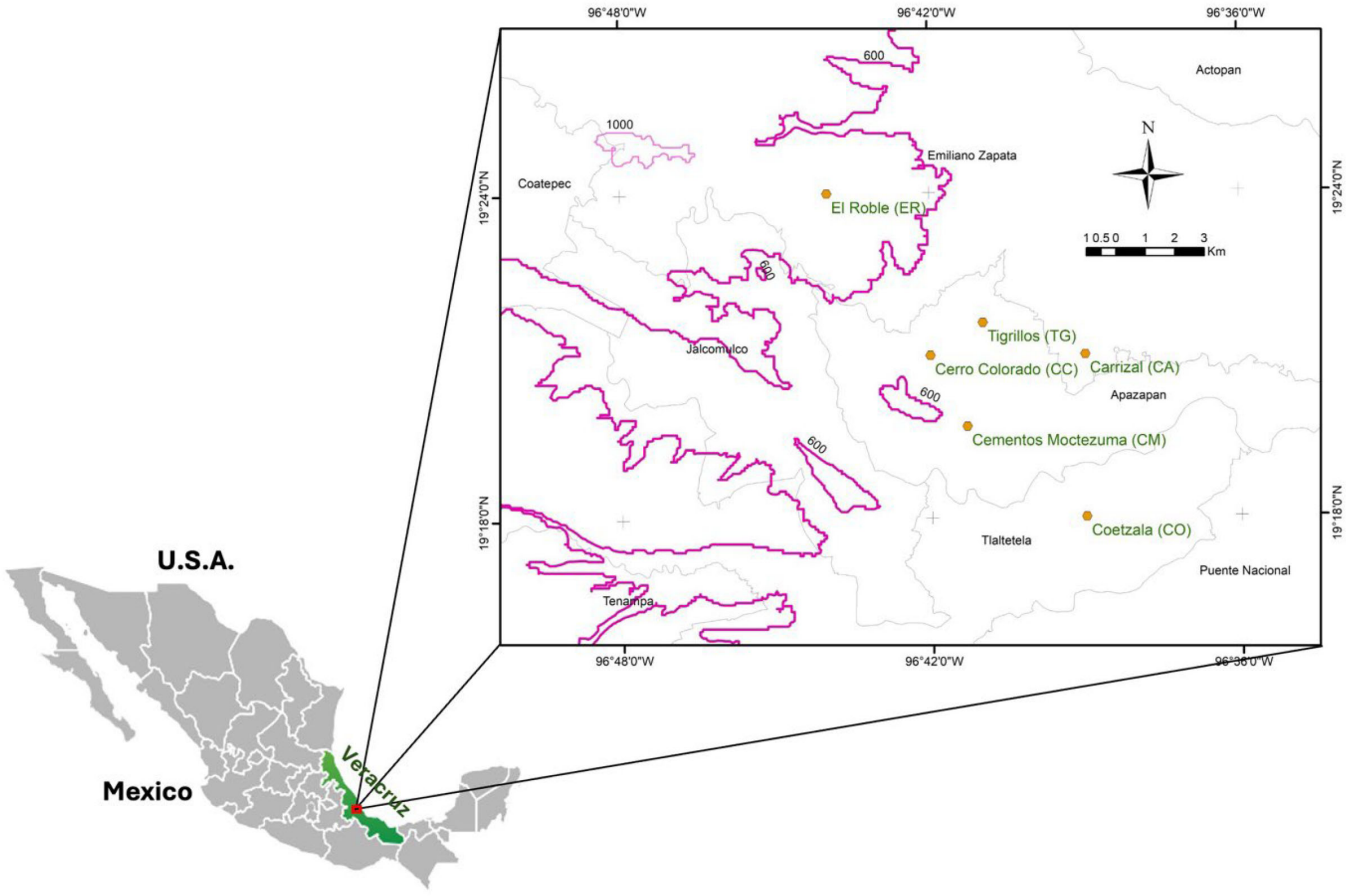
Map of central Veracruz, Mexico, showing the study region and sampling locations. The purple dashed line denotes the 600 m a.s.l. contour, and the gray dashed lines represent municipal boundaries. All sites except El Roble occur below 600 m a.s.l. within natural areas.

Trees of *S. celastrinum* were in rural areas across the municipalities of Emiliano Zapata, Apazapan, and Tlaltetela in the state of Veracruz, Mexico (**Figure 1**). A sampling transect was established during three yearly fruiting seasons (2021 to 2023), encompassing six locations along an elevational gradient as follows: El Roble (ER; 19°24’ N, 96°44’ W; 720 m asl), Cerro Colorado (CC; 19°21’ N, 96°42’ W; 525 m), Tigrillos (TG; 19°22’ N, 96°41’ W; 470 m), Carrizal (CA; 19°21’ N, 96°39’ W; 420 m), Cementos Moctezuma natural area (CM; 19°19’ N, 96°42’ W; 377 m), and Coetzala (CO; 19°18’ N, 96°39’ W; 270 m) (**Table 1**). Site selection followed ecological criteria, considering elevational variation and accessibility to preserved remnants of natural vegetation where the host plant occurs. These parameters ensured spatial and ecological representativeness across the study area.

**Table 1:** Study sites along an elevational gradient in Veracruz, Mexico, where *Sideroxylon celastrinum* was sampled for fruit fly infestation. Site codes, GPS coordinates, mean annual temperature and precipitation and elevation (m.a.s.l. meters above sea level) are shown.

Sites	Code	Geographical Location °N, °W	Temperature and precipitation (Mean annual)	Elevation m.a.s.l.
El Roble	ER	19°24’ N, 96°44’ W	22.02 °C y PP 3.04mm	720
Cerro Colorado	CC	19°21’ N, 96°42’ W	–	525
Tigrillos	TG	19°22’ N, 96°41’ W	–	470
Carrizal	CA	19°21’ N, 96°39’ W	–	420
Cementos Moctezuma	CM	19°19’ N, 96°42’ W	–	377
Coetzala	CO	19°18’ N, 96°39’ W	25.08 °C PP 2.37mm	270

Mature fruits were collected using funnel-type traps installed directly beneath the *S. celastrinum* canopy. Each trap consisted of French tergal fabric (1.5 m × 1.5 m) with a central 1-L plastic container for collecting fallen fruits. One trap was placed on each tree, which was then georeferenced. A total of 45 traps were deployed on 45 individual trees, with sampling efforts depending on the availability of site locations (10 trees in ER, 5 in CA, 8 in TG, 10 in CC, 6 in CM, and 6 in CO).

### Laboratory fruit processing and insect rearing

Collected fruits were transported to the Bioassay Laboratory at the Instituto de Biotecnología y Ecología Aplicada (INBIOTECA, UV), where they were kept until adult insect emerged. During May–June, all fruits were kept at ambient temperature in Xalapa city, under an average temperature of 21.59 ± 0.29 °C (mean ± SE), with recorded maximums of 26.7 ± 0.22 °C and minimums of 16.5 ± 0.12 °C.

From each locality, a random sample of 30 fruits was selected to measured fruit morphology and quantify larvae infestation levels. From this sample, infested fruits were subsequently isolated individually to allow identification of the emerging fly species, these fruits were placed in individual compartment rearing trays (Catalog #8530B, BioQuip Products, Inc.) containing sterilized river sand at the bottom as pupation substrate. The remaining fruits were placed on mesh trays positioned over plastic pans containing 200–250 g of moist sterilized river sand. Each sample was labeled with its tree number and locality of origin. Pupae obtained were transferred to 250-ml plastic containers with moist sterilized river sand and covered with breathable cloth until adult emergence. To assess pupal size for each species, a total of 270 pupae were weighed. Each pupa was subsequently placed in an individual compartment (BioQuip) with moistened sterilized river sand until adult emergence and species identification. Adult fruit flies were identified by VHO (INECOL). Reference specimens of fruit flies were preserved in 70% alcohol and deposited in INECOL and INBIOTECA, while parasitoid samples were deposited in INBIOTECA.

### Data analysis

For each group of fruits sampled per tree at each site, infestation levels were estimated by year, based on the total number of pupae recovered from the samples. The infestation index for each fly species was calculated by dividing the number of adults emerged from the pupae by the total number of adults emerged of both species and multiplying the result by 100. Each fruit used to determine species identity was individually isolated, and all analyses were conducted at the level of individual fruits. All morphological measurements and infestation counts represent per-fruit values rather than aggregated or pooled samples.

Differences in fruit weight and width between infested and non-infested fruits were evaluated separately for *A. dentata* and *A. pallens* using independent two-sample t-tests. The differences between fruits infested with *A. dentata* and *A. pallens* were also analyzed using independent two-sample t-tests to compare mean fruit weight and width, which allowed assessment of species-specific infestation patterns. A 6 × 2 contingency table was constructed using the frequency counts of emerged adults of *A, dentata* and *A. pallens* across sampling sites. Pearson’s chi-square test was applied to evaluate the hypothesis of independence between species identity and sampling site (20). The analysis was conducted using observed frequency counts, whereas percentages (shown in parentheses in **Table 2**) are provided for descriptive purposes only. Because species identity could only be determined for individuals that successfully emerged, non-emerged pupae could not be assigned to species. Therefore, analyses involving emergence success (emerged vs. non-emerged) by species were not possible. Accordingly, our analysis focused on differences in species composition of emerged adults across sites.

**Table 2:** Frequency counts of emerged adults of *Anastrepha dentata* and *A. pallens* across sampling sites (percentages in parentheses). Differences were evaluated using Pearson’s chi-square test based on frequency counts (6 × 2 contingency table).

Sites	*Anastrepha dentata*	*Anastrepha pallens*
El Roble	1059 (74.32)	366 (25.68)
Cerro Colorado	0	0
Tigrillos	40 (34.19)	77 (65.81)
Carrizal	14 (9.79)	129 (90.21)
Cementos Moctezuma	3 (0.90)	330 (99.10)
Coetzala	17 (0.49)	3483 (99.51)
Pearson’s χ^2^ = 3490.40, p < 0.001		

All locality coordinates were georeferenced and converted from degrees–minutes–seconds (DMS) to decimal degrees (DD) using gps-coordinates.net. Maps were constructed using ArcMap software (Version 10.6.1).

Canonical discriminant analysis (CDA) was used to identify which fruit traits best discriminated among fruit categories (*A. dentata*–infested, *A. pallens*–infested, and non-infested fruits) (21). Canonical scores (Root 1 and Root 2) were extracted for each fruit and used to visualize group separation in multivariate space and to evaluate which fruit traits contributed most strongly to patterns of infestation.

All continuous variables were checked for normality (Shapiro–Wilk) and homogeneity of variances (Levene); variables were log- or square-root-transformed when necessary (20) and all tests were performed at a significance level of α = 0.05.

## Results

### Fruit infestation and host-use traits

Fruits of *Sideroxylon celastrinum* infested by *Anastrepha dentata* had a mean weight of 0.617 ± 0.030 g, with mean length and width of 10.46 ± 0.19 mm and 9.20 ± 0.20 mm, respectively (n = 72). In comparison, non-infested fruits averaged 0.5574 ± 0.0250 g in weight and measured 10.71 ± 0.22 mm in length and 8.29 ± 0.16 mm in width. Fruits infested by *A. pallens* had a mean weight of 0.560 ± 0.015 g with mean length and width of 10.09 ± 0.10 mm and 8.58 ± 0.10 mm, respectively. For *A. dentata*, fruit width was significantly greater in infested fruits than in non-infested fruits (t = 3.47, p < 0.001), while fruit weight did not differ in response to infestation (t = 1.49, P = 0.14). In contrast, for *A. pallens*, both fruit (t = 3.21, p < 0.01) and fruit width (t = 2.70, P < 0.01) differed significantly between infested and non-infested fruits.

**Figure 2:**
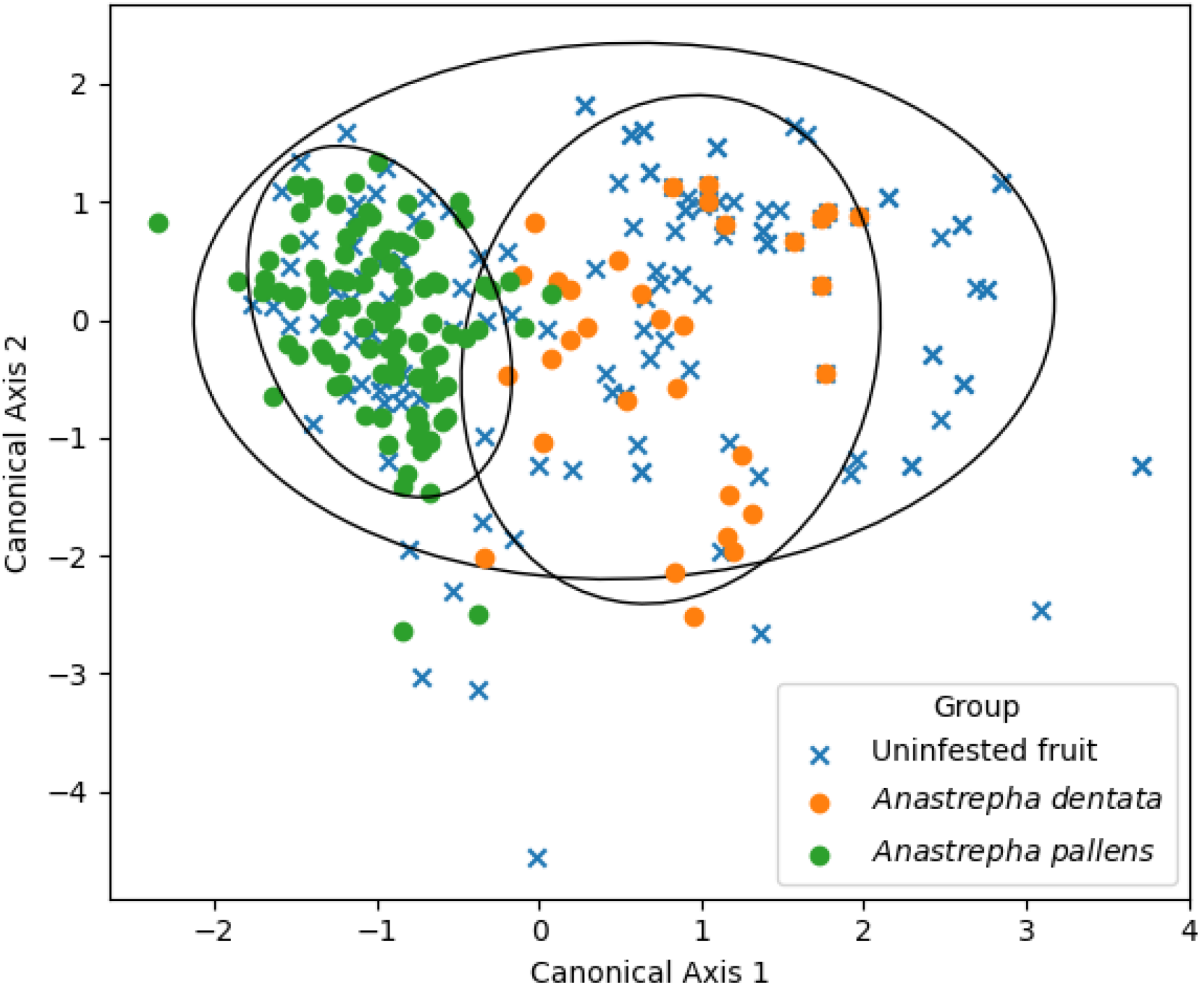
Canonical discriminant analysis of *Sideroxylon celastrinum* fruits classified as *Anastrepha dentata*, *Anastrepha pallens*, or non-infested. Points represent individual fruits plotted using canonical scores (Root 1 and Root 2). Root 1 explained 98.2% of the discriminatory variation and was highly significant (Wilks’ λ = 0.624, χ^2^_6_ = 124.42, p < 0.0001), whereas Root 2 explained little additional variation and was not significant (Wilks’ λ = 0.989, χ^2^_2_ = 2.83, p = 0.24). Colors denote groups: *Anastrepha dentata* (orange), *Anastrepha pallens* (green), non-infested (blue). The ellipses represent the 95% confidence regions for each group, illustrating their distribution and dispersion in multivariate canonical space.

Canonical discriminant analysis extracted two roots, but only the first was statistically significant. Root 1 had an eigenvalue of 0.585 and a canonical correlation of 0.608, accounting for 98.2% of the discriminatory power among groups (Wilks’ λ = 0.624, χ^2^_6_ = 124.42, p < 0.0001) (**Figure 2**). Root 2 was non-significant (Wilks’ λ = 0.989, χ^2^_2_ = 2.83, p = 0.24) and contributed minimally to discrimination (eigenvalue 0.011); thus, it was not retained for interpretation. The structure matrix indicated that fruit length was most strongly correlated with the first canonical function (r = 0.84), followed by fruit width (r = –0.55), whereas weight showed only a negligible association (r = 0.06). Because Root 1 accounted for 98.2% of the discriminating variation and Root 2 was not significant, these correlations indicate that differences in fruit length, with secondary contributions from width, defined the primary morphological axis separating groups. This pattern is consistent with the classification results, where *A. pallens* infested fruits showed greater separation from the other groups, whereas *A. dentata* infested fruits overlapped strongly with non-infested fruits.

### Emergence timing and developmental length

**Figure 3:**
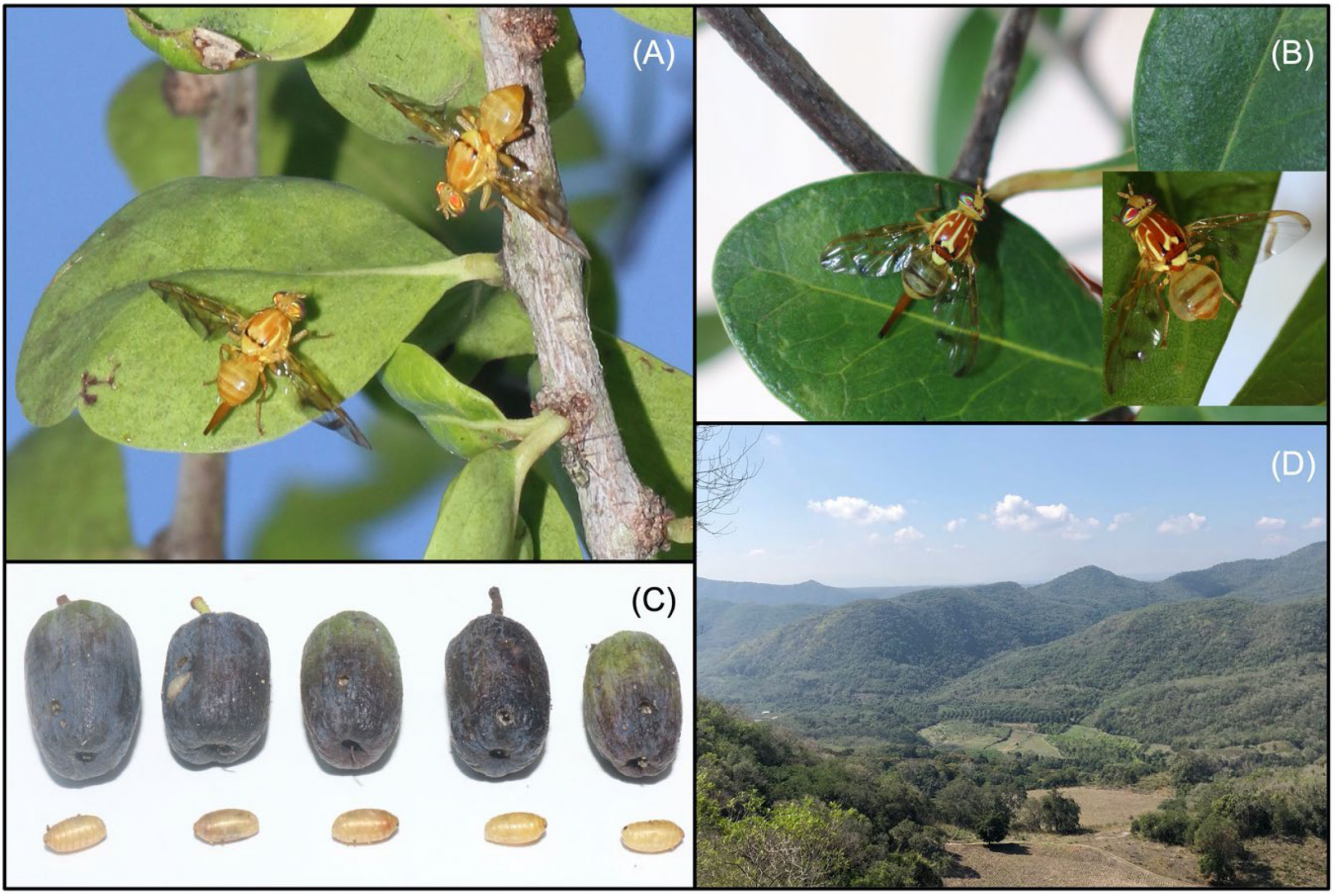
Components of the study system. **(A)** Male (top) and female (bottom) *Anastrepha dentata* on their host plant, *Sideroxylon celastrinum*. **(B)** Female (left) and male (right) *Anastrepha pallens* resting on host foliage. **(C)** Fruits of *Sideroxylon celastrinum* with corresponding pupae of *Anastrepha dentata*, illustrating the one-pupa-per-fruit pattern observed in this system. **(D)** Panoramic view of a representative sampling area between Tigrillos (Apazapan) and El Roble (Emiliano Zapata), Veracruz, showing hilly terrain with fragments of tropical dry forest, intermixed with agricultural plots, orchards, and grasslands.

Adult emergence of *A. dentata* began about 35 days after fruit collection (June) and continued until 60–70 days post-collection, into late July (**Figure 3A**). Emergence of *A. pallens* was earlier and shorter, starting 20 days after collection (May) and concluding 40–45 days later in late June (**Figure 3B**). These differences in emergence timing are consistent with faster development in *A. pallens* under warmer low-elevation conditions (25.08 °C). using similar timing of oviposition by both fly species.

The overall infestation rate was 56.3%, and every infested fruit had only a single larva of either fly species, revealing one-larva–per–fruit occupancy strategy (**Figure 3C**). On average, the pupae of *A. dentata* were significantly heavier (0.258 ± 0.05 mg; n = 60) than those of *A. pallens* (0.174 ± 0.02 mg; n = 180) (t= 2.05, P = 0.042).

### Elevational distribution and species abundance

**Figure 4:**
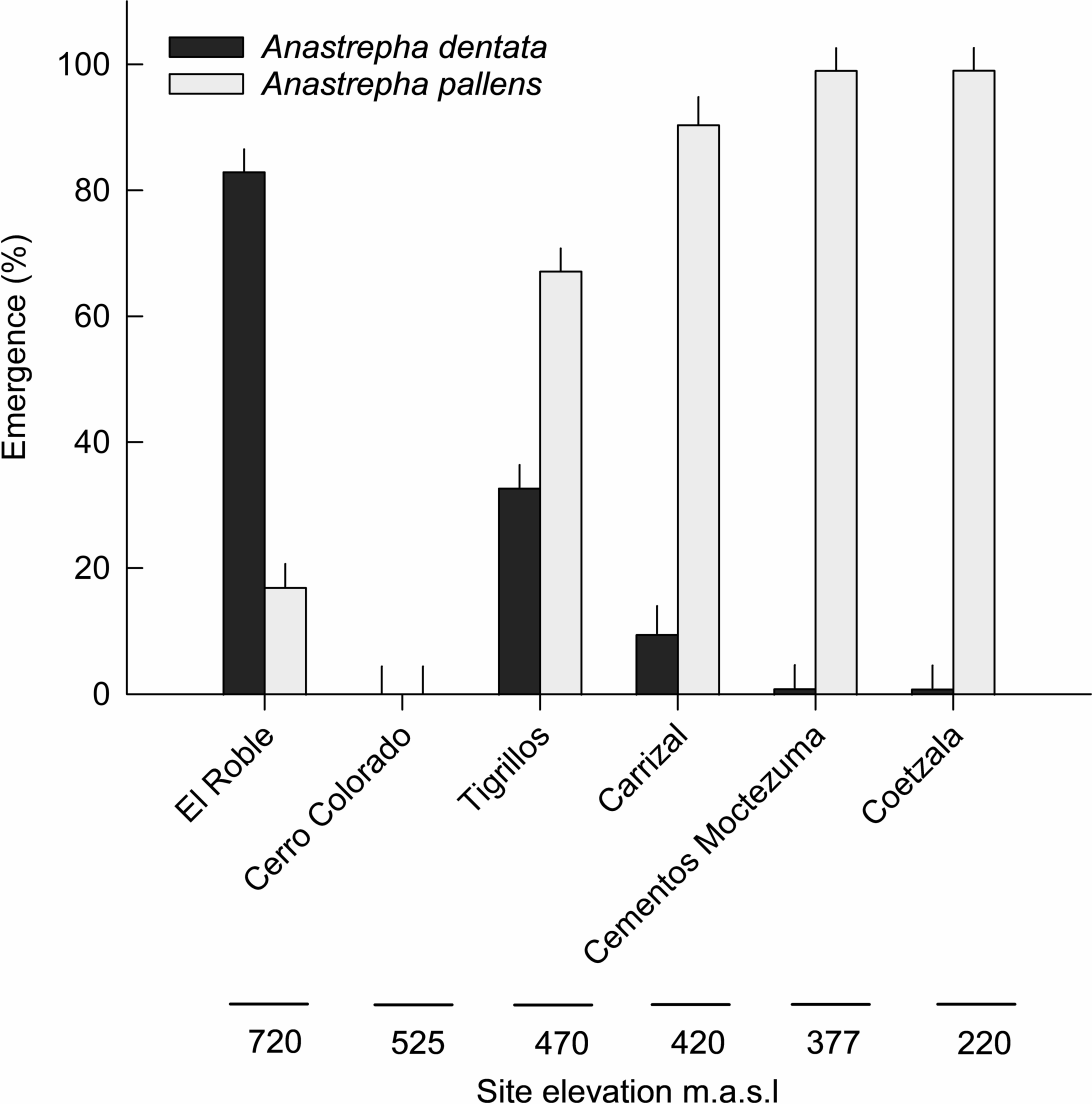
Mean percentage of adult emergence (± SE) from the years 2021–2023 of *Anastrepha dentata* and *Anastrepha pallens* across the six sampling sites. Values illustrate the contrasting elevational distributions of the two species, with *Anastrepha dentata* predominant at higher elevations and *Anastrepha pallens* at lower elevations.

At the higher-elevation site of El Roble (720 m asl), *A. dentata* represented 82.9% of mean adult emergences across the three-year sampling, while *A. pallens* represented 17.1% (**Figure 4**). In 2023, infestation by *A. dentata* reached up to 95% (**Table 3**). In contrast, at lower site Coetzala (270 m asl), this species accounted for < 1% of emergences, while *A. pallens* comprised > 99% (**Table 2**). The intermediate-elevation site of Tigrillos (~470 m asl) exhibited mixed dominance, with *A. dentata* reaching up to 35% of emergences in 2021, suggesting a transition zone where both species can coexist. On the other hand, fruits sampled at Cerro Colorado (525 m asl), were exclusively infested by larvae of Curculionidae (Coleoptera). These seed predators induced premature fruit maturation and abscission, and no *Anastrepha* larvae were detected, despite adequate sampling effort (**Table 3**). This indicates that seed-feeding weevils may limit fruit availability and reduce access of fruit flies to the host resource at this location. Adult emergence patterns of *A. dentata* and *A. pallens* differed significantly across sampling locations, with *A. dentata* emerging predominantly at high-elevation sites, and *A. pallens* dominating lower-elevation areas.

**Table 3:** Infestation levels and biological parameters of *Anastrepha dentata* and *Anastrepha pallens* associated with *Sideroxylon celastrinum*, based on fruit collections during 2021–2023 in Veracruz, Mexico.

Sites	Year	Pupas	*Anastrepha dentata*			*Anastrepha pallens*			Emergence total (%)
			Male	Female	Emergence (%)	Male	Female	Emergence (%*)*	
El Roble	2021	244	77	72	84.2	13	15	15.8	177 (72.5)
	2022	1206	377	355	68.9	178	152	31.1	1062 (88.1)
	2023	229	83	95	95.7	5	3	4.3	186 (81.2)
Cerro Colorado	2021	0	0	0	0.0	0	0	0.0	0
	2022	0	0	0	0.0	0	0	0.0	0
	2023	–	–	–	–	–	–	–	–
Tigrillos	2021	114	19	12	35.6	31	25	64.4	87 (76.3)
	2022	–	–	–	–	–	–	–	–
	2023	38	4	5	30.0	12	9	70.0	30 (78.9)
Carrizal	2021	85	4	2	10.3	26	26	89.7	58 (68.3)
	2022	63	2	3	10.0	25	20	90.0	50 (79.4)
	2023	45	2	1	8.6	14	18	91.4	35 (77.8)
Cementos Moctezuma	2021	211	0	0	0.0	89	83	100.0	172 (81.5)
	2022	134	1	2	3.0	52	44	97.0	99 (73.9)
	2023	87	0	0	0.0	29	33	100.0	62 (71.3)
Coetzala	2021	2801	1	0	0.04	1259	1135	99.96	2395 (85.5)
	2022	948	6	5	1.4	398	349	98.6	758 (80.0)
	2023	470	3	2	1.4	160	182	98.6	347 (73.8)
TOTAL		6675	579	554	20.5	2291	2094	79.5	5518 (82.7)

The contingency table, analyzed using Pearson’s chi-square test, revealed a strong association between fly species and sampling location (χ^2^_4_ = 3490.40, p < 0.001), indicating pronounced geographic structuring in the species composition of emerged adults (**Table 2**). Specifically, *Anastrepha dentata* predominated at lower-elevation sites, whereas *A. pallens* was more abundant at higher elevations. Parasitoid emergence was low overall. Few specimens of *Doryctobracon areolatus* (Viereck) (Hymenoptera: Braconidae) were recovered from only two locations at Coetzala (4 males, 7 females), and El Roble (2 males, 3 females). The host fly species from which the parasitoids emerged could not be determined.

## Discussion

Our work yielded several important and novel findings. Notably, this is the first report on the trophic association of *Anastrepha dentata* (Stone) larvae feeding on fruits of *Sideroxylon celastrinum* (Kunth) T.D. Pennington. We also provide evidence that *A. dentata* and *Anastrepha pallens* (Coquillett) coexist on the same host species, and exhibit differences along an elevational gradient. The cohabitation patterns reveal changes in niche structure based on elevation-mediated niche partitioning, which was reinforced by variations in host development time. Previous reports indicated that *A. dentata* is only known from trapping records in some Mexican states. Conversely, *A. pallens* has been documented in trapping surveys across at least 14 Mexican states, showing a wider geographical and ecological distribution (13).

The genus *Anastrepha* is highly diverse, encompassing species ranging from strict specialists to broad generalists. Field observations and fruit dissections revealed that both species leave a single larva per fruit, which primarily feed on the seeds, suggesting direct competition for the same resource within the fruit. However, their sustained coexistence over a long period of time supports the hypothesis of ecological differentiation, which deserves further investigation.

Furthermore, our research demonstrates that infestation by *A. pallens* was related to fruit size rather than weight, with the former being the main morphological predictor of host use. The canonical discriminant analysis identified fruit length as a more informative variable than fruit weight in distinguishing infestation patterns between *A. dentata* and *A. pallens*. This result is biologically plausible, as fruit length may better reflect oviposition surface area and internal spatial structure, which can influence egg-laying behavior and larval performance. Longer fruits may provide greater separation among larvae, potentially reducing intra- and interspecific competition, as well as offering more favorable microhabitats for larval development. Subtle differences in host use have been observed in other *Anastrepha* trophic interactions, such as the differential use of *Psidium guajava* L. by *Anastrepha fraterculus* (Wiedemann) and *Anstrepha striata* Schiner (8). Similar cases of coexistence on the same host plant among other conspecifics have also been documented (22, 23). Future studies on infestation patterns should therefore incorporate fruit size as a central predictive variable in the exploitation of these resources.

Phylogenetic analyses indicate that species associated with Sapotaceae hosts tend to occupy basal lineages within the genus *Anastrepha* (1). Furthermore, many species in various clades exploit hosts in the Sapotaceae family, suggesting a long-standing use of these hosts (24). The placement of *A. dentata* within the dentata group and *A. pallens* within the daciformis group suggests that exploitation of Sapotaceae fruits may represent an ancestral trophic association retained across multiple clades. The coexistence of these species on *S. celastrinum* therefore provides an opportunity to examine how ecological differentiation emerges among closely related lineages sharing ancestral host resources (1, 12, 24). These groups appear to be highly specialised in feeding on Sapotaceae plants (3). Our results therefore support their close association with seeds, confirming an ancestral evolutionary pattern with the Sapotaceae family.

Differences in pupal weight between species suggest different developmental strategies. The heavier pupae found in *A. dentata* could be indicative of greater investment in larval development, resulting in larger adults; and pupal emergence occurring later (35–70 days after collection) suggests a slower developmental cycle in colder higher-altitude environments. In contrast, *A. pallens* showed earlier emergence (20–45 days), indicative of faster development in warmer, lower-altitude conditions. Therefore, the observed differences in emergence timing may reflect both species-specific developmental rates and variation in the timing of oviposition events in the field. However, the exact developmental stage of larvae at the time of collection could not be determined.

The density of infestation varied significantly along the altitudinal gradient. At El Roble (720 m), *A. dentata* was the dominant species, accounting for 95% of infestations. In contrast, at Coetzala (270 m), *A. pallens* accounted for over 98% of emergences. At intermediate altitudes (Tigrillos), however, both species coexisted in the fruits. Infestations ranged from 30-35% for *A. dentata*, while *A. pallens* accounted for 64.3-70% of adult recovered in the samples. Our findings support the hypothesis that altitudinal distribution is mediated by microclimatic factors, such as temperature and humidity, which are the main determinants of species distribution. This phenomenon aligns with the broader ecological premise that environmental heterogeneity promotes the coexistence of related species (25) and is consistent with the altitude partitioning observed in *Anastrepha*-*Psidium* interactions (26, 27).

There were no infestations by *Anastrepha* larvae in Cerro Colorado (525 m) since the fruits were exclusively infested by an undetermined species of Curculionidae. Although we cannot conclusively determine whether these beetles directly exclude fruit flies, their seed-feeding behaviour and induction of premature fruit drop could reduce the availability of oviposition resources for *Anastrepha*. This idea is supported by the existence of indirect competition mechanisms for resources (28, 29). Our observations suggest that the presence of third-party herbivores could influence host availability, thereby affecting trophic interactions.

The detection of the wasp parasite *Doryctobracon areolatus* (Szépligeti) in Coetzala and El Roble indicates a functional, albeit low-intensity tritrophic interaction whithin the *Anastrepha*–*S. celastrinum* system. However, the low parasitism rates prevented an assessment of species-specific susceptibility. These low rates may be related to the intrinsic features of Sapotaceae fruits such as latex (30), as well as the location of the larvae within the seeds, which may limit access by parasitoids. Notably, Sivisnki et al. (31) reported that ovipositor length is more closely related to preferences and adaptations to specific environments and ecological niches. Nevertheless, these mechanisms were not addressed here and require further investigation. Notably, *D. areolatus* has been widely reported as the most prevalent parasitoid in interactions between *Anastrepha* species and their host plants (32, 33), implying broad ecological tolerance and adaptability to many fruit fly species.

A thorough understanding of host-specific preferences is essential for accurately predicting population dynamics and responses to environmental variations. According to Bjørnstad et al. (34), synchronous fluctuations in subpopulations could increase the risk of local or regional extinction. Cohabitation patterns between *A. dentata* and *A. pallens* revealed elevation-mediated niche partitioning, which was reinforced by variations in host development time. Our results support the hypothesis of ecological diversification in *Anastrepha*, emphasising the pivotal role of environmental factors such as altitude and temperature, in shaping the trophic interactions and population structure of these systems.

The study confirms that *S. celastrinum* serves as a host plant for *A. dentata* which coexists with *A. pallens*, both feeding on the seeds. Despite sharing this resource, the two species were found to be segregated by elevation. *Anastrepha pallens* dominated in the lowlands, while *A. dentata* was mainly found at higher elevations. We also proved that contrary to what was expected, fruit size rather than weight was the main morphological predictor of infestation by *A. pallens*, since larger fruits were most heavily infested. These findings improve our understanding of the trophic interactions between *Anastrepha* species and their native host plants. Niche partitioning and host specialisation within the Sapotaceae family provide insight into the broader ecological processes shaping *Anastrepha* communities in the Neotropics.

## Data Availability

The raw data supporting the conclusions of this article will be made available by the authors, without undue reservation.
